# Durability of Metal-Composite Friction Spot Joints under Environmental Conditions

**DOI:** 10.3390/ma13051144

**Published:** 2020-03-04

**Authors:** Seyed M. Goushegir, Nico Scharnagl, Jorge F. dos Santos, Sergio T. Amancio-Filho

**Affiliations:** 1 Solid State Joining Processes, Materials Mechanics, Institute of Materials Research, Centre for Materials and Coastal Research, Helmholtz-Zentrum Geesthacht, 21502 Geesthacht, Germany; mgoushegir@gmail.com (S.M.G.); jorge.dos.santos@hzg.de (J.F.d.S.); 2 Corrosion and Surface Technology, Magnesium Innovation Center (MagIC), Materials Mechanics, Institute of Materials Research, Centre for Materials and Coastal Research, Helmholtz-Zentrum Geesthacht, 21502 Geesthacht, Germany; nico.scharnagl@hzg.de; 3 BMVIT Endowed Professorship for Aviation, Institute of Materials Science, Joining and Forming, Graz University of Technology, 8010 Graz, Austria

**Keywords:** friction spot joining, fiber reinforced composites, aluminum alloys, aging, outdoor environmental durability, mechanical properties

## Abstract

The current paper investigates the durability of the single-lap shear aluminum-composite friction spot joints and their behavior under harsh accelerated aging as well as natural weathering conditions. Four aluminum surface pre-treatments were selected to be performed on the joints based on previous investigations; these were sandblasting (SB), conversion coating (CC), phosphoric acid anodizing (PAA), and PAA with a subsequent application of primer (PAA-P). Most of the pre-treated specimens retained approximately 90% of their initial as-joined strength after accelerated aging experiments. In the case of the PAA pre-treatment, the joint showed a lower retained strength of about 60%. This was explained based on the penetration of humidity into the fine pores of the PAA pre-treated aluminum, reducing the adhesion between the aluminum and composite. Moreover, friction spot joints produced with three selected surface pre-treatments were held under outside natural weathering conditions for one year. PAA-P surface pre-treated specimens demonstrated the best performance with a retained strength of more than 80% after one year. It is believed that tight adhesion and chemical bonding reduced the penetration of humidity at the interface between the joining parts.

## 1. Introduction

Metal-composite hybrid structures have been gaining more attention lately from the transport industry. Recently alternative joining techniques, such as Friction Spot Joining (FSpJ), have been developed to join lightweight metals with polymer composites. FSpJ was shown to be a reliable joining process for joining metals with thermoplastic-based composites [1,2,3,4,5]. In our previous publications, we investigated the influence of various process parameters on the mechanical performance of the FSp joints [3,5]. Moreover, the effect of different aluminums’ surface pre-treatments on the lap shear strength of the aluminum alloy 2024-T3/carbon-fiber-reinforced poly (phenylene sulfide) (CF-PPS) FSp joints was recently reported [6], as well as its impact resistance [7] and corrosion properties [8] for Al-sand-blasted treatment.

In addition to initial strength, the durability of a joint, which is its ability to retain initial strength under harsh environments for long time, is particularly important for metal-composite hybrid structures [9], because engineering structures such as an airplane or car are constantly exposed to the environment. In order to select and use a specific joining method, its long-term behavior must be understood. Usually, due to time limitations, the aging of a joint is analyzed over a shorter time, but under an extremely harsh environment (high relative humidity and high temperature). This is known as accelerated aging.

It is important to understand the degradation mechanisms under accelerated aging conditions, to be able to design a durable joint. Three types of mechanisms may cause the degradation of a metal-polymer joint in a humid environment.

The first mechanism is the degradation of the metal-polymer interface [10,11,12]. If the metal-polymer bonding contains weak boundaries, where no intimate contact exists as a result of poor surface wetting, moisture may diffuse at the interface. Moisture can degrade the adhesion forces such as hydrogen bonds [10], leading to a reduction of joint strength and durability.

The second mechanism to consider is the influence of moisture on the polymer [13,14]. It has been suggested that humidity may degrade the properties of the polymer through plasticization [13,14,15] or the generation of swelling stresses [13,14]. Thus, a weakening of the polymer is another reason for the reduced durability of a joint. In addition to the humidity, ultraviolet (UV) radiation is another source of degradation for polymers and composites. It is frequently reported [16,17] that photo-oxidation, as a result of UV radiation, changes the physical properties of polymers, such as discoloration and increase in the glass transition temperature, and reduces their mechanical performance. Such behavior should also be considered when using polymers and composites in a structure.

The third mechanism suggests the degradation of the metallic part, in this case aluminum [18,19]. It is well known that aluminum oxide is prone to hydration in a humid atmosphere [19,20]. Aluminum hydroxide forms a weak layer that may be easily detached from the underlying aluminum oxide. Aluminum oxide converts into the crystalline aluminum hydroxide (AlOOH) known as boehmite [16,18]. Upon further hydration, AlOOH transforms into Al(OH)_3_, known as bayerite [13,21]. Accordingly, the hydration of the aluminum surface also degrades the joint durability.

Sealants and paints may be used as a solution against the above-mentioned durability degradation mechanisms. To reduce the hydration of the aluminum surface, various surface pre-treatments may also be useful. Electrochemical pre-treatments showed the highest durability, followed by chemical and mechanical pre-treatments in adhesively bonded aluminum joints [22,23]. The durability of sandblasted joints was reported to be better or inferior to chemically pre-treated bonded joints in different studies reviewed in [23]. This might be attributed to the extent of the macroporosity generated on the aluminum surface and the wettability of the surface. Proper wettability is necessary for obtaining a durable joint when mechanical pre-treatment is employed. Electrochemical pre-treatments showed excellent durability as a result of the generation of a thick oxide layer, forming a barrier against humidity and corrosive environments.

Among the electrochemical pre-treatments, it is reported that phosphoric acid anodizing (PAA) offers the best durability [18]. Davis et al. explained for the first time the mechanisms of hydration inhibition by PAA pre-treatment [20]. They pointed out that a very thin layer of AlPO_4_ is formed on top of the aluminum oxide. This layer absorbs water from humidity to form AlPO_4_·H_2_O, which inhibits the further hydration of the underlying aluminum oxide. Nevertheless, if the aluminum is exposed to a humid atmosphere sufficiently long, the AlPO_4_·H_2_O layer starts to dissolve. This leaves the underlying oxide layer exposed to moisture and it begins to degrade.

To further protect the aluminum against hydration and corrosion, a suitable primer layer may be used. Bland et al. used an epoxy-based primer containing strontium and chromium particles on a PAA pre-treated aluminum alloy prior to adhesive bonding [22]. Their findings suggest that the primed joint had a better durability compared to PAA pre-treatment alone.

It is clear from the explanations above that a proper surface pre-treatment not only enhances the adhesion mechanisms and therefore initial joint strength but also the durability of the joint. No information could be found in the literature regarding the influence of surface pre-treatments on the accelerated aging behavior of welding-based joining techniques. A few works have been published aiming at understanding the mechanical performance of metal-polymer hybrid joints under natural outside weathering [24,25]. Didi et al. investigated the influence of different aluminum surface pre-treatments on the mechanical performance of AA5754/carbon-fiber-reinforced polyamide 66 (CF-PA66) induction welded joints after one year of weathering conditions [24]. The authors reported that degreasing and corundum blasting resulted in a very low retained lap shear strength after 12 months. By using acid etching and combined corundum blasting and acid etching, the retained strength increased to more than 50% and 60% respectively. Recently, Schricker et al. demonstrated a strength reduction of approximately 50% in AA6082 / PA66 laser joints after 12 weeks of natural weathering [25]. However, the authors claimed that the mechanical performance also depends on the selected joining speed. Such a reduction in strength was attributed to the moisture absorption and plasticization of PA66.

The current paper deals with the durability of single-lap shear (SLS) FSp joints and their behavior under harsh accelerated aging as well as natural weathering conditions. Various surface pre-treatments were applied on the surface of aluminum to investigate their influence on the failure and mechanical performance of the joints. Besides mechanical characterization, different microscopy and analytical techniques such as scanning electron microscopy (SEM), energy-dispersive X-ray spectroscopy (EDS), and X-ray photoelectron spectroscopy (XPS) were employed to analyze the surface of the joints as well as fracture surfaces after mechanical testing to evaluate the influence of the aging condition on the joints.

## 2. Experimental Section

### 2.1. Materials

Aluminum alloy AA2024-T3 rolled sheets with a 2 mm thickness (Constellium, Paris, France) were selected as the metallic part in this work. This alloy is mainly used in transport applications, particularly in aircrafts. Fatigue resistance and damage tolerance, high toughness, and a high strength to weight ratio are some of the main properties offered by AA2024-T3 [26].

As the composite part, CF-PPS laminated sheets (supplied by TenCate, Nijverdal, the Netherlands) with a 2.17 mm nominal thickness were used. The sheets consisted of five harness woven quasi-isotropic laminates with seven plies of carbon fibers [(0.90)/(±45)]3/(0.90). Furthermore, 50 vol % (42 wt %) of continuous carbon fibers was used in this composite. CF-PPS is a high-performance semi-crystalline thermoplastic composite with main applications in primary and secondary aircraft parts. It offers high strength, rigidity, chemical resistance, and low water absorption [27,28,29].

### 2.2. FSpJ Process

FSpJ was used in this work to join the parts together. The principles of the process have been explained in our previous publications [1,2,3,4,6]. Briefly, the process uses a non-consumable tool, plunging into the aluminum sheet, which was placed on top of the composite in an overlap configuration to a pre-defined position while rotating at high speed. As a result of the plunging of the rotating tool into the aluminum sheet, frictional heat is generated around the tool. Thereby, a volume of the aluminum under the tool is deformed (known as the metallic nub) and inserted into the composite due to the applied axial force by the tool. The metallic nub creates a mechanical interlocking between the joining parts, especially under shear loading. At the same time, the frictional heat is conducted to the interface between the aluminum and composite. As a result, a thin layer of the composite’s matrix melts, which after solidification (during the cooling phase) generates adhesion forces between the joining parts. For more information on the process and bonding mechanisms, refer to the previous publications.

Friction spot joints were produced using position-controlled equipment (RPS 100, Harms&Wende, Hamburg, Germany). An optimized set of joining parameters (tool rotational speed: 2900 rpm, tool plunge depth: 0.8 mm, joining time: 4 s, and joining pressure: 0.3 MPa) was selected to join the single lap shear specimens based on the previous investigations [5]. Specimens from AA2024-T3 and CF-PPS were machined prior to the joining process with dimensions of 100 × 25.4 mm. An overlap area of 25.4 × 25.4 mm was selected to join the specimens. The surface of the aluminum samples was treated before joining. Four surface pre-treatments were selected; these were sandblasting (SB), stand-alone conversion coating (CC), phosphoric acid anodizing (PAA), and PAA with a subsequent application of primer (PAA-P). Although SB + CC gave a slightly higher lap shear strength than stand-alone CC in dry conditions [6,30], the CC specimen was selected for the aging experiments, in order to understand the behavior of chemical pre-treatment under environmental conditions. For a detailed explanation of each surface pre-treatment, refer to [6,30].

### 2.3. Accelerated Aging

To investigate the behavior of the FSp joints under harsh environments, the SLS FSp joints of the selected aluminum surface pre-treatments were placed in an artificial aging chamber (VCL 0003, Vötsch Industrietechnik, Balingen, Germany) for 28 days. The temperature of the chamber was set at 71 °C with 100% relative humidity following the recommendations given in the ASTM D3762 standard [31]. From the conditions given in the ASTM standard, the environment selected for this work was the most severe one. The humidity of the chamber during the test was constantly controlled and adjusted by pumping water into the chamber. After 28 days, the joints were removed from the chamber for further analysis. In addition to the mechanical testing and chemical composition measurements, the samples were weighed to measure the moisture uptake. The samples were first exposed to the stream of air at 40 °C for 1 h before measuring their weight.

### 2.4. Weathering Conditions

In addition to the accelerated aging, a set of samples with selected aluminum surface pre-treatments was held under outside natural weathering conditions (Geesthacht, Germany) for one year during the period of December 2013 to December 2014. During the exposed year, the air temperature, relative humidity, precipitation, and wind speed were monitored. The SLS specimens were removed in two intervals, after six months and one year, for further mechanical testing.

### 2.5. Microscopy

SEM (Quanta^TM^ FEG 650 equipment, ThermoFisher Scientific, Houston, TX, USA) was used to analyze the surface of the aluminum samples and the fracture surface of the joints after mechanical testing. To analyze the surface of the aluminum specimens, a voltage of 10 kV, spot size of 3, and a working distance of 10 mm were used. In the case of the fracture surfaces, a voltage of 5 kV, spot size of 3, and a working distance of 15 mm were set. Before analyzing non-conductive samples (e.g., all the fracture surfaces), their surfaces were gold-sputtered using a Q150R ES equipment (Quorum Technologies Ltd., Lewes, UK) for 30 s with a current of 65 mA.

### 2.6. EDS and XPS

EDS coupled with SEM was carried out to investigate the chemical changes on the surface of the aluminum after accelerated aging. To obtain and analyze the EDS spectra, an EDAX TEAM^TM^ software V4.0.2 (Edax Inc., Mahwah, NJ, USA) was used. Both spot and area analyses were used to characterize small features and larger areas respectively. All EDS spectra were taken with a voltage of 10 kV, spot size of 3, at a working distance of 10 mm. For the non-conductive specimens, gold sputtering was performed prior to the EDS experiments. For those specimens, a gold peak is thereby present in the respective spectra.

Furthermore, XPS was used to confirm changes of the aluminum oxide layer after the accelerated aging process. For that, a Kratos DLD Ultra Spectrometer (Kratos Analytical Ltd., Manchester, UK) with an Al-Kα X-ray source (monochromator) operated at 225 W was selected. For the region scans, a pass-energy of 40 eV was chosen. Charge neutralization was performed for all specimens. The calibration of the spectra of contamination-free surfaces was performed to a 284.8 eV binding energy of the C1s signal. CasaXPS V.2.3.16 software (Casa Software Ltd., Teignmouth, UK) was used to process the data.

### 2.7. Single Lap Shear (SLS) Testing

All SLS specimens after accelerated aging and weathering conditions were mechanically tested under tensile loading according to the ASTM D3163-01 standard [32], using a universal testing machine (model 1478, Zwick Roell, Ulm, Germany) with a load capacity of 100 kN. A traverse test speed of 1.27 mm/min was selected, and the tests were performed at room temperature. Five replicates were tested to obtain the average ultimate lap shear force (ULSF) of the joints.

## 3. Results and Discussion

The results of this work are separated in two parts. In the first part, the results obtained from the accelerated aging conditions are discussed. In the second part of the paper, the mechanical performance of the joints under outdoor weathering conditions is briefly addressed. It should also be noted that the influence of different surface pre-treatments on the bonding mechanism and mechanical performance of the FSp joints was discussed thoroughly in [30]. Briefly, SB and PAA treatment led to a rough aluminum surface, increasing the micromechanical interlocking between aluminum and the molten polymer. Conversion coating altered the chemical state of the aluminum surface on a nanoscale and enhanced the chemical (covalent) bonding. Finally, PAA-P led to strong primary bonding between the primer layer and the molten PPS.

### 3.1. Accelerated Aging

#### 3.1.1. Surface Features and Chemical Composition

**Figure 1:**
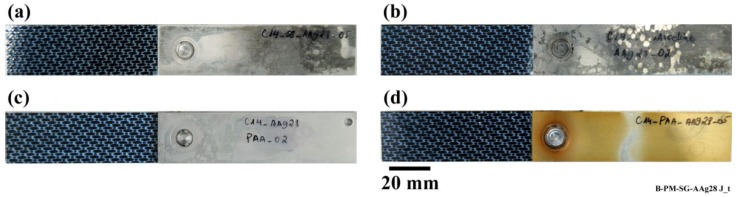
Top view of the SLS FSp joints after 28 days of aging; (**a**) SB, (**b**) CC, (**c**) PAA, and (**d**) PAA-P.

**Figure 2:**
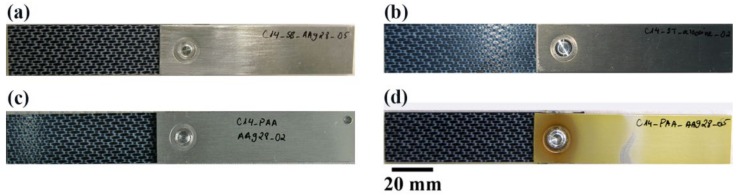
Top view of the SLS FSp joints prior to aging; (**a**) SB, (**b**) CC, (**c**) PAA, and (**d**) PAA-P.

First of all, the joints were visually inspected as soon as they were taken out of the aging chamber. Figure 1 shows the top view of the SLS FSp joints after 28 days of the aging experiment, and Figure 2 the same joints before aging. Noticeable changes could be seen in the SB and CC pre-treated specimens on the aluminum part. Dark regions were identified both on the top and bottom surfaces of the SB and CC pre-treated aluminum. Aluminum oxide, formed on the surface of the SB and CC pre-treated specimens, interacts with the humidity in the aging chamber, which leads to the formation of a weak aluminum hydroxide layer. It is well known that an aluminum surface undergoes hydration in the presence of a high level of humidity or when immersed in water [33]. Despite the PAA and PAA-P samples having slight water stains on the aluminum, no notable changes could be identified. PAA pre-treatment is known to produce an oxide layer that is more corrosion resistant than CC [34]. This could be the reason that the PAA pre-treated specimen did not exhibit any noticeable surface changes after 28 days of aging. Moreover, phosphate ions in the AlPO_4_ monolayer that is formed on the aluminum surface after PAA pre-treatment reduce the hydration rate of the aluminum, as reported in [20,35]. On the PAA-P specimen, the primer is a thick, corrosion resistant layer [36,37,38], which inhibits the interaction of the underlying aluminum oxide with humidity. That is why no visual changes could be observed on the PAA-P sample. Finally, the composite parts did not show any visual changes after 28 days of aging. This was expected, because PPS is a highly moisture-resistant polymer [29].

**Figure 3:**
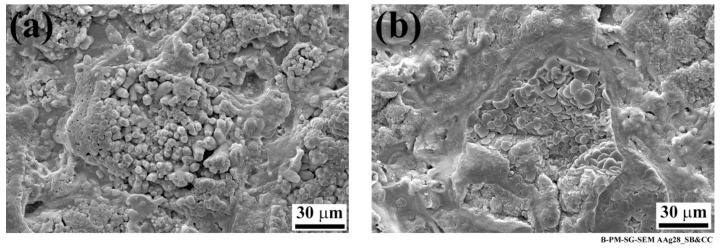
High-magnification SEM images of the aluminum affected areas after 28 days of aging; (**a**) SB and (**b**) CC specimens showing nodular, flake-like structures.

To further analyze the aluminum surfaces, high-magnification SEM images were taken from the affected areas on the specimens. Both SB and CC specimens showed compact areas consisting of the very fine nodular and flake-like structures that are related to the weak aluminum hydroxide formation (Figure 3).

Table 1 shows the average chemical composition of the aluminum in the affected areas for the SB and CC pre-treated specimens, obtained from the EDS analysis. The results reveal that Al and O are the main elements present in these areas. A small amount of carbon was also detected on both specimens, which may be related to contamination in the aging environment. An even smaller amount of N was identified on the CC specimen, also from the humid environment in the aging chamber. The results showed an enormous increase in oxygen compared to the specimens before aging (see Table 2). In both cases, the oxygen content increased by more than five times after accelerated aging. This increase in oxygen was reported due to the conversion of aluminum oxide into hydroxide [19]. In contrast to the as-pre-treated specimens, other AA2024-T3 alloying elements, such as Cu and Mg, were not detected on the aged aluminum surfaces. Such an alteration of elements on the aged surfaces confirms the formation of a thick aluminum hydroxide layer on the SB and CC pre-treated specimens.

**Figure 4:**
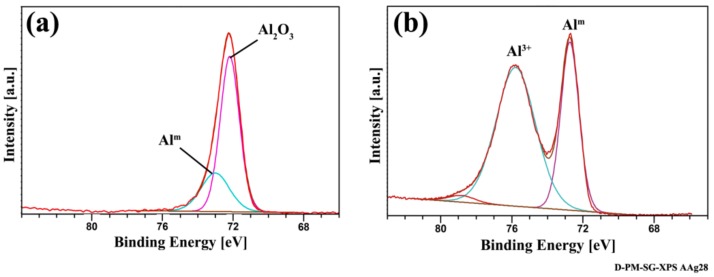
High-resolution Al 2p XPS region spectra of the SB specimen (**a**) before aging and (**b**) after aging.

Furthermore, an XPS analysis could further confirm the conversion of the oxide layer into aluminum hydroxide. Figure 4 shows a high-resolution Al 2p region of the SB specimen before and after accelerated aging. The aluminum before aging (Figure 4a) revealed two peaks at approximately 72 eV and 72.8 eV that are related to aluminum oxide [39,40,41] and metallic aluminum [39,42,43] respectively. After aging, the peak at 72.8 eV (related to the metallic aluminum) was still detectable, but the peak at 72 eV disappeared, and a new peak at approximately 76 eV was identified. The appearance of this peak might be due to the aluminum hydroxide formation [41]. It was reported that aluminum oxyhydroxide (AlOOH) is the most common form of aluminum hydroxide generated on the aluminum surface in the presence of humidity and at a temperature range of 25–100 °C [44,45,46,47]. However, it has also been suggested that, after further aging, the hydration of the AlOOH leads to the formation of Al(OH)_3_ [21]. Regardless of the type of aluminum hydroxide present, the hydration of aluminum oxide was confirmed through an XPS analysis.

**Figure 5:**
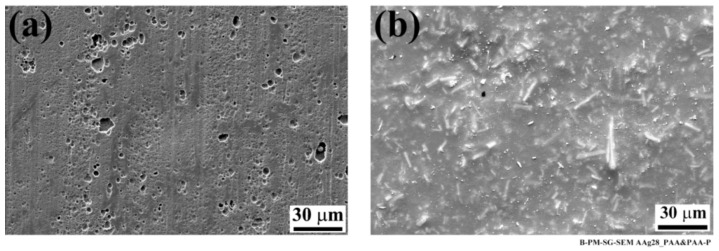
SEM images of the aluminum side of the joint after 28 days of aging; (**a**) PAA and (**b**) PAA-P specimens.

**Figure 6:**
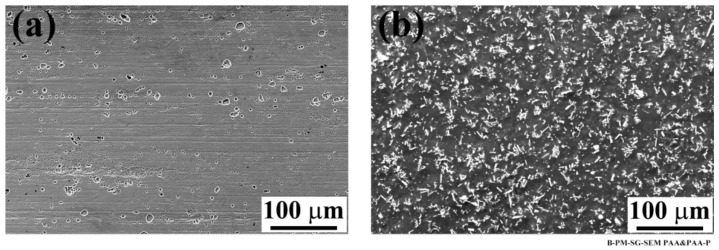
SEM images of the pre-treated aluminum surface (**a**) PAA and (**b**) PAA-P specimens. Reproduced with permission from [30].

In contrast to the SB and CC specimens, the PAA and PAA-P samples did not show any noticeable changes on the aluminum surface after aging, as visually compared in Figure 1 and Figure 2. The SEM images of the aluminum surface, illustrated in Figure 5, appear very similar to the ones before aging (Figure 6). The PAA specimen (Figure 5a) showed an open porous structure with some coalesced pores, similar to its surface before aging (Figure 6a). The compact structure of the PAA-P specimen was also retained with the whisker-like particles of chromium and strontium oxides, as shown in Figure 5b.

As with the SB and CC specimens, an EDS analysis was performed on the PAA and PAA-P specimens. The results are listed in Table 3. In addition to Al, O, and C, in contrast with the SB and CC specimens, Cu and Mg were identified on the surface of the PAA specimen. The identified elements are very similar to those before aging (Table 4), with the exception of P, which was not detected after aging. The results reveal that the only major alteration of the PAA surface after aging is a reduction in aluminum concentration by about 7 wt % and an increase in carbon content by approximately 10 wt %. The increase in carbon content could be attributed to contamination from the aging chamber and the humid environment itself. Such an increase in carbon content as a new layer on the aluminum surface would slightly reduce the aluminum content captured by the EDS analysis. Furthermore, it was suggested that the hydration of the PAA pre-treated aluminum surface starts with a slow dissolution of the AlPO4 layer, followed by the conversion of the aluminum oxide into aluminum hydroxide [20,33,48]. The absence of the P in the EDS analysis may be correlated with the early stages of the hydration process.

The behavior of the PAA-P specimen was slightly different to the PAA sample. The EDS analysis of the PAA-P pre-treated aluminum after aging (Table 3) showed similar elements to the one before aging (Table 4), with the addition of an N peak. However, the quantification of the elements, as listed in Table 3 and Table 4, revealed that the carbon content was reduced by 15 wt %, whereas the aluminum content showed an increase of approximately 8 wt %. This clearly indicates a thickness reduction of the carbon-based primer layer leading to a reduced carbon content. Moreover, the aluminum beneath the primer layer could be detected in a higher concentration due to a reduced primer thickness. In addition, a 10 wt % reduction of the oxygen content after aging was also identified. Since there were various sources of oxygen, the aluminum oxide, primer, chromium, and strontium oxides, a partial removal of the primer layer appears to have more influence on the reduction of the oxygen content. Finally, both Cr and Sr showed an increase in the content of approximately 7 wt % and 10 wt % respectively after aging. This is probably due to the partial removal of the carbon contained in the primer layer (as a result of its interaction with humidity), leading to an exposure of chromium and strontium oxides. Therefore, a higher concentration of the whisker-like oxides could be observed.

#### 3.1.2. Mechanical Performance of the SLS Joints

**Figure 7:**
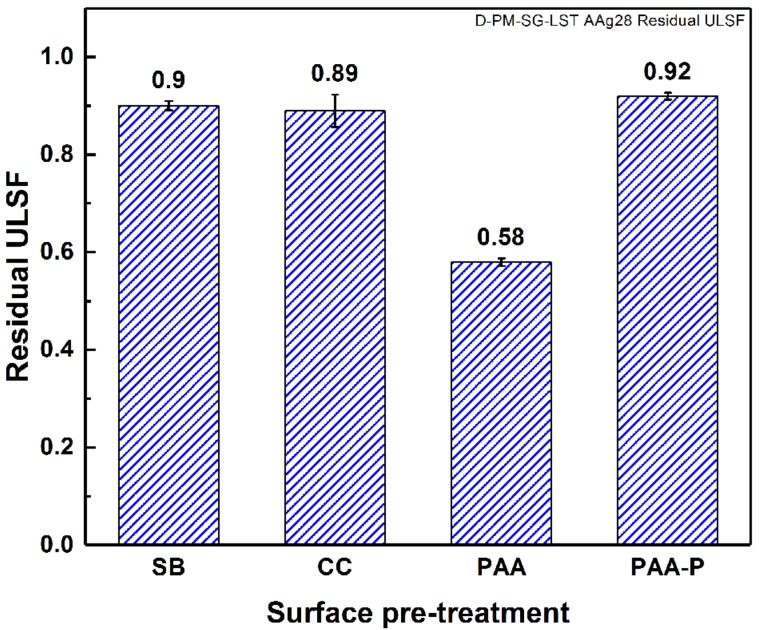
Residual strength of the SLS FSp joints after 28 days of accelerated aging.

The joints were mechanically tested to evaluate their lap shear strength shortly after the removal of the joints from the aging chamber (within 1 h). The obtained lap shear strengths of the SLS joints were divided by their initial strength before aging, and the results were reported as the residual strength of the joints, as illustrated in Figure 7. The SB, CC, and PAA-P specimens had only a small reduction in strength, but the PAA specimen was approximately 42% reduced, compared to their initial strength. Such results are in agreement with those reported in the literature for adhesively bonded aluminum joints, for example in [49].

**Figure 8:**
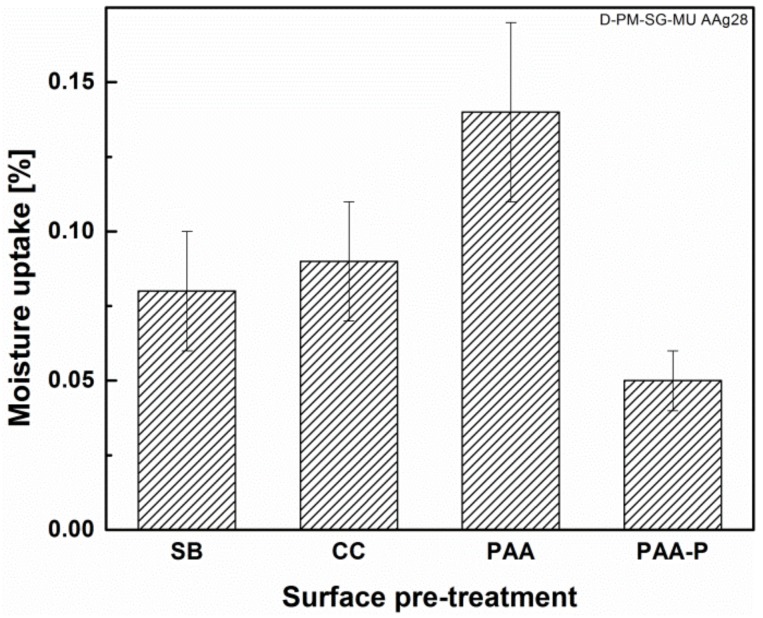
Moisture uptake of the FSp joints after 28 days of accelerated aging.

It is believed that both the morphology and chemical composition of the aluminum surface play important roles in the durability of FSp joints. The SB pre-treatment generated large pores and crevices on the surface of the aluminum that could be filled almost completely by the molten PPS throughout the bonding area. Since molten PPS wet and fill such crevices, moisture cannot penetrate easily or rapidly into the interface between the aluminum and PPS, which in turn reduces the degradation kinetic of the joints. Furthermore, the chemical bonding between the aluminum and PPS, in the cases of CC and PAA-P pre-treatments [30], reduces the moisture path into the joints. In all three cases, the moisture diffusion was not completely inhibited, but the diffusion kinetic was significantly reduced. This is the reason for the small reductions in strength, compared to the initial strength of the joints. By contrast, it seems that the moisture could penetrate more easily and much more rapidly with the PAA pre-treated joint, leading to aluminum-PPS interface degradation and hence a reduction in mechanical performance. Figure 8 demonstrates the amount of moisture uptake of the FSp joints after the aging time. One observes that the PAA pre-treated joints showed the highest moisture uptake, approximately twice that of the SB pre-treated samples. The moisture was absorbed primarily at the interface between the aluminum and composite, degrading the bonding between the parts and hence the mechanical performance of the FSp joints. The larger reduction of the lap shear strength of the PAA pre-treated joints can therefore be related to the higher moisture uptake and humidity penetration into the bonding area.

**Figure 9:**
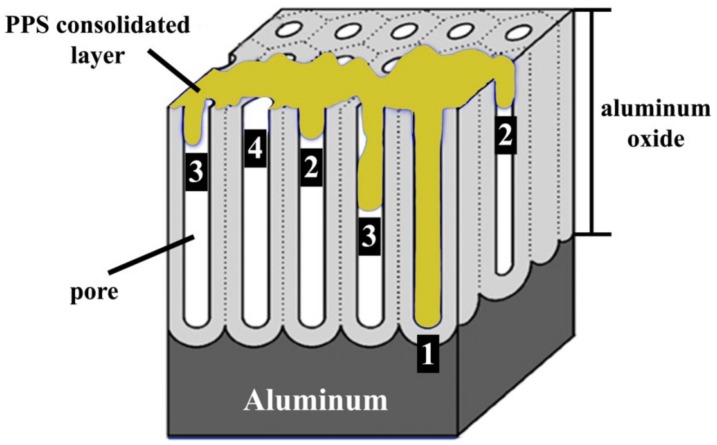
Schematic illustration of aluminum oxide after PAA pre-treatment, adapted from [51], and the proposed model of pore filling by the PPS. (**1**) Complete wetting and pore filling, (**2**) complete wetting, incomplete pore filling, (**3**) partial wetting and incomplete pore filling, and (**4**) no wetting and no pore filling.

In the PAA pre-treated specimen, the diffusion of moisture into the interface may be related to the morphology of the oxide layer formed and the extent of pore filling by the PPS. A model with four possible situations for aluminum oxide pore filling by PPS is proposed here, as illustrated in Figure 9. The four possible pore filling cases can be summarized as follows:

(1) Complete wetting and pore filling
(2) Complete wetting, incomplete pore filling
(3) Partial wetting or pore filling
(4) No wetting or pore filling.

**Figure 10:**
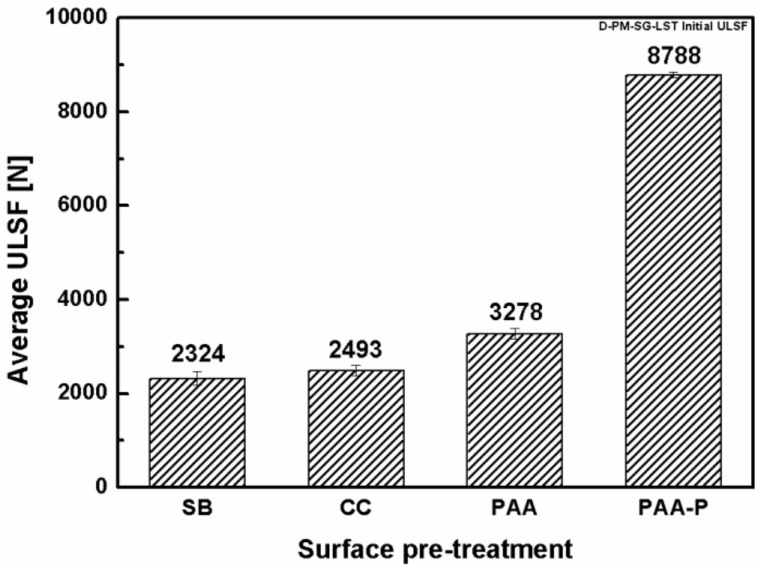
Initial strength of the SLS FSp joints before accelerated aging.

Incomplete pore filling (Cases 1 and 2) could still result in adequate micro-mechanical interlocking and an acceptable initial strength. The initial strength of the PAA specimens was higher than for the SB specimens (Figure 10) because of the presence of a much larger amount of pores, which could be filled (partially or completely) by the molten polymer. However, such incomplete pore filling is detrimental to the durability of the joints. According to the proposed model, while the joint is in contact with a humid atmosphere, the diffusion of the humidity into the interface depends on the pore filling situation. In Case 1 and Case 2, where the wetting between pore walls and the PPS is complete, the humidity diffusion is expected to be sluggish. By contrast, in Case 3 and particularly in Case 4 the humidity can penetrate much faster into the pores and into the interface of the aluminum-PPS. This leads to the degradation of the interface and hence the mechanical strength of the joint. These results are in agreement with the theories reported in the literature. Kinloch et al. suggested that in adhesively bonded aluminum, interfacial micro-voids in PAA pre-treated aluminum allow for the penetration of the water (or humidity) into the interface between the aluminum and adhesive [35]. The penetration of water was reported to be detrimental to the durability of the adhesive joint. Moreover, Digby and Packham stated that, in adhesive bonding, obtaining durable joints depends on the penetration of the adhesive into the aluminum oxide pores [50]. Such a penetration was considered to be dependent on several factors, such as the pore dimensions, adhesive viscosity, and the viscosity characteristic of the adhesive at a working temperature. Incomplete pore filling was reported to be the main reason for the reduced durability of the joints for specific surface pre-treatments such as PAA [50]. Therefore, it is believed that complete wetting and pore filling, as well as strong chemical bonds, are important aspects in achieving durable FSp joints.

#### 3.1.3. Failure and Fracture Surface Analysis

**Figure 11:**
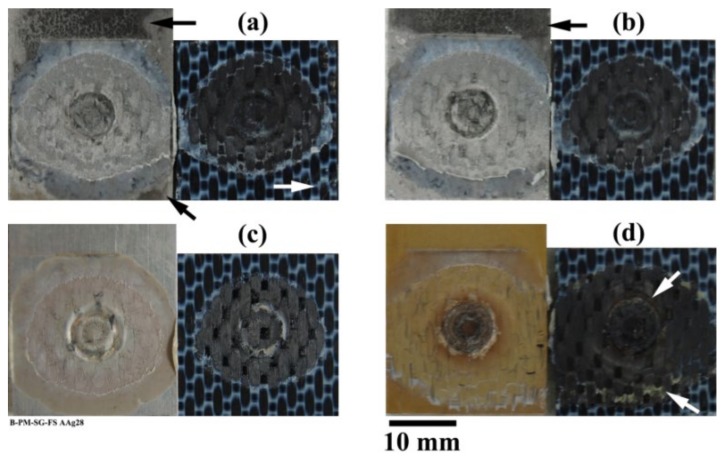
Fracture surface of the SLS joints after 28 days accelerated aging; (**a**) SB, (**b**) CC, (**c**) PAA, and (**d**) PAA-P. The black arrows in (**a**) and (**b**) indicate the aluminum hydroxide formation. The white arrow in (**a**) indicates small features outside the AZ. The white arrows in (**d**) indicate the primer remaining attached to the CF-PPS.

Figure 11 shows the fracture surface of the four pre-treated joints after 28 days of accelerated aging. The dark aluminum hydroxide layer can be observed on the SB and CC pre-treated specimens even very close to the consolidated molten PPS (known as the Adhesion Zone (AZ) [4,52]), as indicated by the black arrows in the figure. However, in none of the joints could any indication of aluminum hydroxide formation inside the bonding area be detected. In FSpJ, strong micro-mechanical interlocking and/or adhesion forces between the aluminum and consolidated molten PPS significantly reduce the moisture diffusion into the Plastically Deformed Zone (PDZ) [4,52], the area inside the consolidated molten PPS. Therefore, the rate of the interface deterioration is reduced, as was observed from the residual strength of the joints, shown in Figure 7. Although, the PAA specimen did not show any significant changes on the fracture surface (Figure 11c), humidity diffusion was expected to take place faster than with the other surface pre-treatments, as was also explained by the moisture uptake. The PAA-P specimen also showed very similar features to the specimen before aging (refer to [30]), such as the primer remaining attached to the composite, as indicated by the white arrows in Figure 11d.

**Figure 12:**
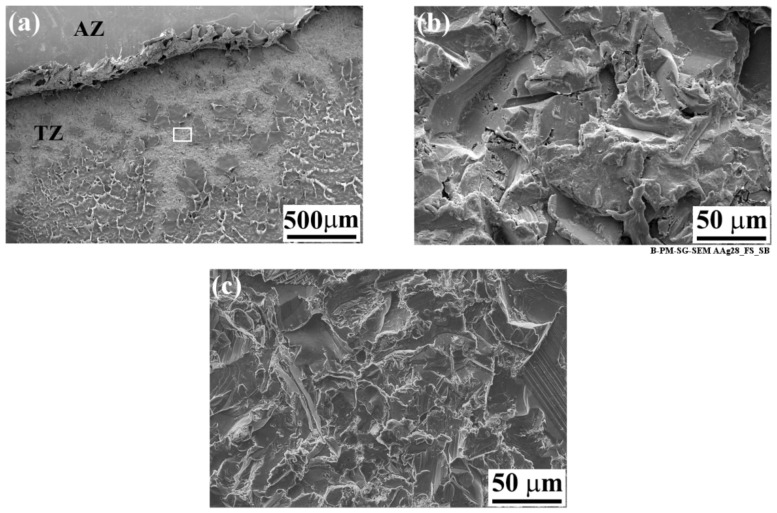
SEM image of the fracture surface of the SB specimen on the aluminum side after 28 days of accelerated aging. (**a**) Low-magnification image of the AZ-TZ area, (**b**) high-magnification image of the white rectangle indicated in (**a**), and (**c**) high-magnification image of the TZ area prior to aging.

Figure 12a shows the fracture surface of the SB specimen on the aluminum side of the joint. The image illustrates the AZ with a very smooth surface at the top of the image followed by a Transition Zone (TZ) [4,52]. The TZ shows typical features, where PPS remains attached to the aluminum as individual islands [52]. Figure 12b is a high-magnification image of the exposed SB aluminum indicated by the white rectangle in Figure 12a inside the TZ. No obvious alteration of the aluminum surface could be identified when compared to the SB surface before aging (see Figure 12c). This confirms that a large amount of moisture did not penetrate inside the bonding area, nor did it convert aluminum oxide into aluminum hydroxide in the bonding area. The same hypothesis seems to be valid for the rest of the surface pre-treatments, with the exception of PAA.

**Figure 13:**
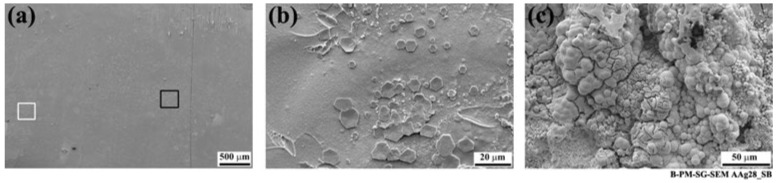
SEM image of the fracture surface of the SB specimen on the composite side after 28 days of accelerated aging. (**a**) Low-magnification image from the area indicated by the white arrow in Figure 11a; (**b**) high-magnification image from the black rectangle indicated in (**a**), and (**c**) high-magnification image from the white rectangle indicated in (**a**).

As indicated by the white arrow in Figure 11a, small features could also be observed on the composite side of the fracture surfaces outside the AZ. As an example, Figure 13 shows such features on the CF-PPS of the SB specimen. Although the low-magnification image (Figure 13a) did not reveal any specific features, the high-magnification images (Figure 13b,c) show flake-like features and agglomerates of small particles. As this area on the CF-PPS corresponds to the aluminum hydroxide on the aluminum side of the joints, it is believed that these particles are the hydroxide layer removed from the aluminum and remaining attached to the composite. Despite the fact that these particles were outside the bonding area, they remained attached to the composite. This may be attributed to the weak nature of the hydroxide layer, which was easily detached from the underlying aluminum oxide as a result of frictional forces between the aluminum and composite during the lap shear testing of the joint.

The EDS analysis further confirmed that both the flake-like structures and the agglomerates of particles contained Al, Cu, Mg, and O, as listed in Table 5. In both cases, the presence of aluminum and a high amount of oxygen, when compared to the as-pre-treated specimen, indicated that these particles were aluminum hydroxide. Sulfur from the underlying PPS could be detected in the case of the flake-like structures, which was an indication of the thinness of the flakes. However, because the agglomerates were larger in thickness, no sulfur from the PPS was detected in the respective EDS spectrum.

### 3.2. Outdoor Natural Weathering

**Figure 14:**
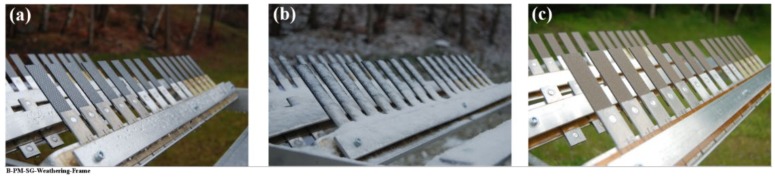
Outdoor natural weathering of the AA2024-T3/CF-PPS friction spot joints; specimens (**a**) during the first month, (**b**) during the third month, and (**c**) during the sixth month showing the discoloration of the composite.

In addition to the accelerated aging, the SLS specimens were placed outside in natural weathering conditions for one year, as can be seen in Figure 14. Note that in this case three aluminum surface pre-treatments were selected: SB, CC, and PAA-P. PAA pre-treated specimens were not included as a result of their lower performance during accelerated aging experiments. Different climate data were recorded in this time frame, as follows [53]: an average temperature between −12.9 °C and 33 °C; a relative humidity of 3% to 100%; an average precipitation (both rain and snow) of 16.3 mm to 112.2 mm per month; an average UV index between 1 and 6; and a wind speed of 0.4 km/h to 62.6 km/h.

The aluminum/composite FSp joints were influenced both by humidity and moisture absorption as well as UV irradiation. CF-PPS showed discoloration as a result of the UV irradiation, as demonstrated in Figure 14c. It was reported by Batista et al. that such discoloration of CF-PPS is due to photolysis and photo-oxidation, resulting in an increase in the glass transition temperature [16]. Furthermore, surface embrittlement of the composite was observed as a result of the extensive cross-linking of polymer chains [16].

It should also be noted that the effect of wind speed may not be neglected, since one end of the joints was not clamped and additional loads could be exerted on the specimens.

#### Mechanical Performance of the SLS Joints

**Figure 15:**
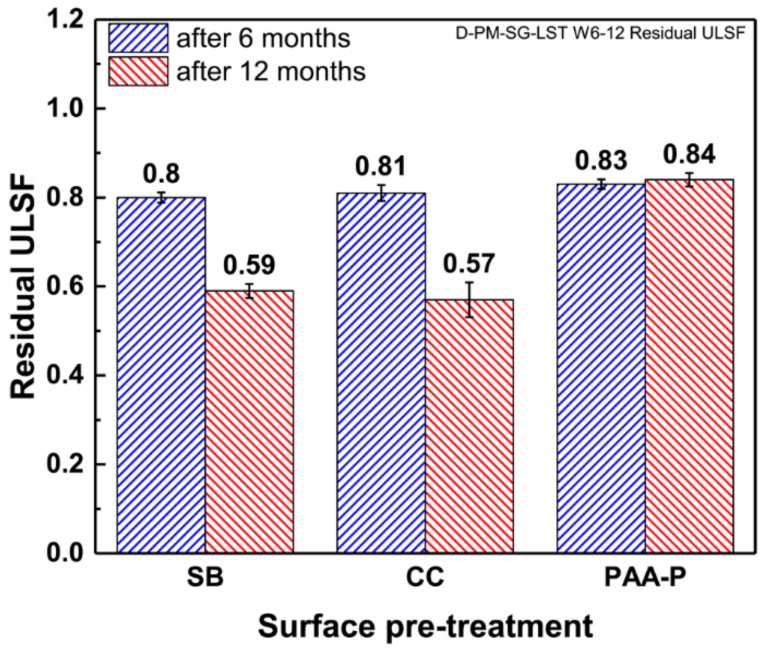
Residual strength (ULSF) of the SLS FSp joints after six and 12 months of outdoor weathering.

The specimens were mechanically tested in two time intervals: six and 12 months. The obtained lap shear strengths of the SLS joints were divided by their initial strength prior to weathering, and the results were reported as the residual strength of the joints, as illustrated in Figure 15. Both SB and CC specimens showed a similar trend. In the first six months a reduction of 20–30% in the lap shear strength was observed. The trend of reduction in strength was also observed in the next six months. However, after one year both sets of joints retained more than 50% of their initial lap shear strength. These results are similar to those reported for the induction welding of the metal-composite joints after weathering conditions [24].

**Figure 16:**
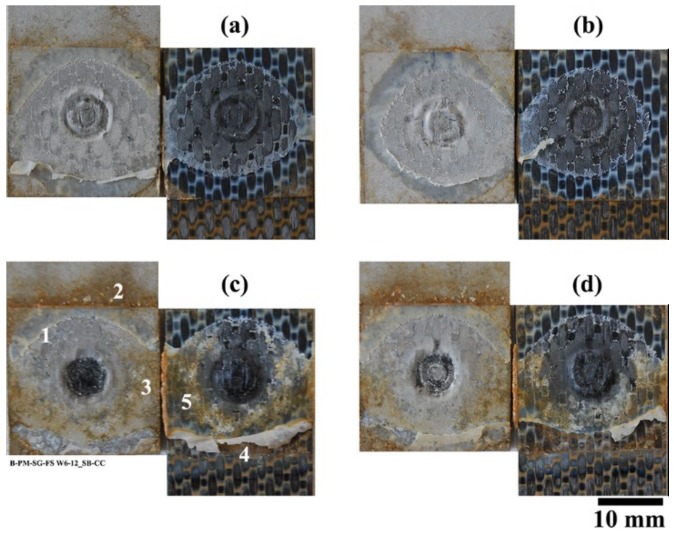
Fracture surface of the pre-treated joints after outdoor weathering. (**a**) SB and (**b**) CC after six months, (**c**) SB and (**d**) CC after 12 months.

It can be argued that the moisture and humidity could penetrate slightly into the overlap area, deteriorating the bonding between the aluminum and composite, especially in the area of the consolidated molten PPS (known as AZ). Because AZ is the weakest part of the bonding area in a FSp joint [4,6,52], the penetration of the moisture inside this layer should be easier. As the fracture surface of the joints shows in Figure 16a,b, no visual changes were observed within the bonding area of SB and CC specimens after six months. Despite the lack of apparent changes on the fracture surfaces, it is valid to argue that the penetration of the moisture into the bonding area may happen slowly, which could weaken the bonds on the microscale. Moreover, one should consider the fact that the precipitation in areas near the ocean contains salty elements. Such elements have indeed a different (perhaps harsher) influence on the aluminum-polymer bonds rather than a pure humid atmosphere (as in the accelerated aging experiments). In contrast to the first six months, the fracture surfaces of the SB and CC joints after 12 months demonstrated the penetration of the moisture and humidity into the bonding area, as shown in Figure 16c,d. That is the reason for the further decrease in the residual strength of the joints.

The clear distinction between the fracture surface of the specimens after six and 12 months (both for SB and CC) is that after 12 months a large amount of the consolidated molten PPS remained attached to the composite. This is in contrast with the fracture surface of the joints prior to the weathering and those after six months of outdoor weathering, in which the consolidated layer was attached almost fully to the aluminum.

**Figure 17:**
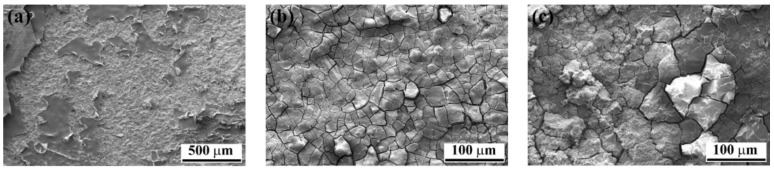
SEM images of the fracture surface of the SB specimen on the aluminum side after 12 months of outdoor weathering. (**a**) TZ from the area (1) in Figure 16c; (**b**) high-magnification image from the area (2) in Figure 16c, and (**c**) high-magnification image from the area (3) in Figure 16c.

The SEM analysis of the fracture surface on the aluminum side reveals interesting features, as shown in Figure 17. The TZ from the unaffected region (Figure 17a) shows similar features (PPS remained attached to the aluminum as individual islands and a sandblasted aluminum surface) to the joints before weathering. However, the aluminum surface from the affected regions (Figure 17b,c) demonstrates a very cracked surface. The phenomenon is similar to the intergranular corrosion of the aluminum 2xxx alloys [54]. Since the precipitation (particularly in areas near the ocean, where this work was carried out) may contain small amounts of sodium and chloride ions [55], corrosion may have slightly occurred in these specimens.

**Figure 18:**
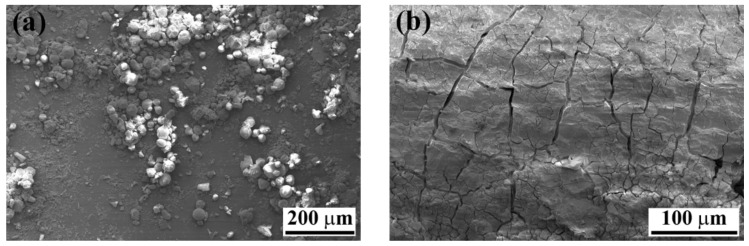
SEM images of the fracture surface of the SB specimen on the composite side after 12 months of outdoor weathering. (**a**) high-magnification image from the area (4) in Figure 16c, and (**b**) high-magnification image from the area (5) in Figure 16c.

Furthermore, the SEM images from the composite side (Figure 18) show similar features as for the aluminum surface. Spherical features were observed on the composite outside the bonding area (Figure 18a), whereas a cracked surface was detected inside the bonding area (Figure 18b).

**Figure 19:**
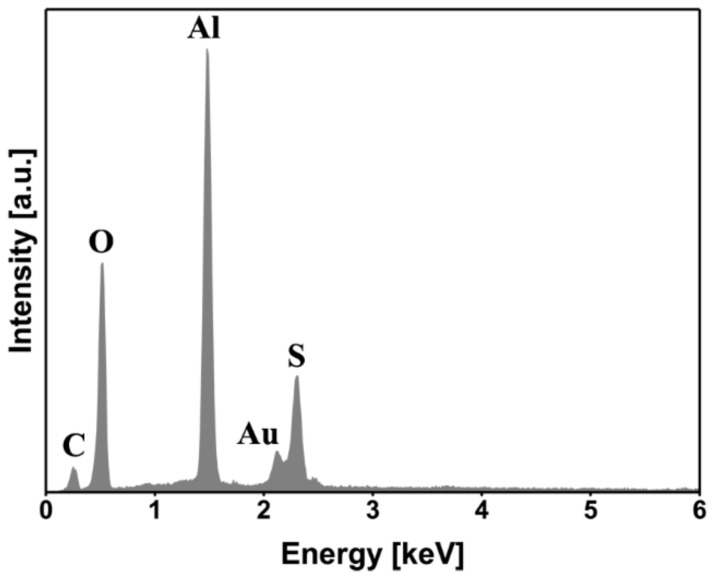
EDS area analysis of the fracture surface shown in Figure 18b.

The EDS analysis of the cracked surface in Figure 18b on the composite side is illustrated in Figure 19. In addition to sulfur and carbon (from the PPS), aluminum and oxygen peaks were also detected. This confirms that the cracked region is the aluminum, which remained attached to the composite. It is believed that the aluminum was slightly corroded starting outside the bonding area and penetrated beneath the PPS consolidated molten layer. One may identify the corroded/aged aluminum outside the bonding area as a weak point. During the mechanical testing, cracks may initiate from this weak corroded layer and propagate inside the aluminum in the bonding area. This is the reason why a part of the aluminum remained attached to the consolidated molten PPS on the composite side. Therefore, the lower mechanical strength of the SB and CC joints after 12 months of natural aging is believed to be the result of the weakening of the aluminum alloy rather than the bonding area.

**Figure 20:**
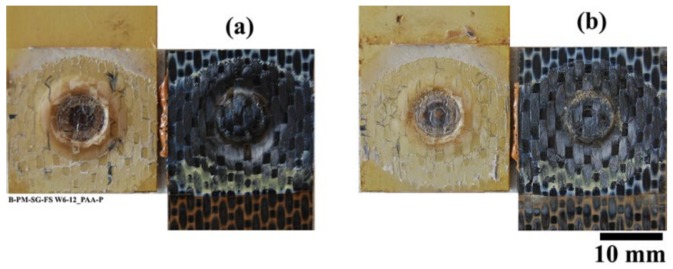
Fracture surface of the PAA-P pre-treated joints after (**a**) six months and (**b**) 12 months of outdoor weathering.

In contrast to the SB and CC specimens, the PAA-P pre-treated joints showed a reduction in the lap shear strength in the first six months, while retaining their residual strength on the same level afterwards. PAA-P pre-treatment led to strong chemical carbon-carbon bonds between CF-PPS and aluminum [6,30]. It seems that moisture does not have a great influence on such chemical bonds, and the joint retained more than 80% of its initial strength. Moreover, the fracture surface of the PAA-P joints did not reveal any apparent changes as a result of the moisture penetration in the bonding area, as demonstrated in Figure 20.

It is worth noting that, although CF-PPS showed discoloration due to the UV irradiation, no changes in color could be observed inside the overlap area, as illustrated in Figure 16 and Figure 20. Therefore, it is not expected that UV irradiation had any significant effect on the deterioration of the mechanical strength of the joints.

These results suggest that a suitable surface pre-treatment not only increases the initial strength of the metal-composite joint but also enhances the long-term durability under environmental conditions.

## 4. Conclusions

An accelerated aging experiment was carried out on four selected surface pre-treatments: SB, CC, PAA, and PAA-P. The surface of the aluminum outside the bonding area, after 28 days of aging, showed a dark layer on the SB and CC specimens. This dark layer was determined to be Al(OH)_3_ aluminum hydroxide, as confirmed by EDS and XPS analyses. In contrast with the SB and CC specimens, the PAA and PAA-P samples did not have any noticeable changes on the aluminum surface. Although the SB and CC specimens showed the formation of a weak aluminum hydroxide layer, the residual strength of these joints was approximately 90% of the initial dry quasi-static strength. This was comparable with the residual strength of the PAA-P pre-treated joint, which was 92% of the initial dry quasi-static strength of the respective joint. The high residual strength of the SB, CC, and PAA-P pre-treated joints was ascribed to the low level of moisture diffusion in the bonding area. Moisture diffusion was significantly decelerated due to the favorable wetting of the aluminum surface by the molten PPS. In contrast, PAA pre-treated joints resulted in a residual strength of approximately 58% after accelerated aging. This may be explained by the partial wetting and pore filling of the aluminum oxide layer by the molten PPS. A very fine structure of the pores, a high viscosity of the PPS, and a very fast cooling rate are the main causes of the partial wetting. Such partial wetting allows for moisture diffusion, degrading the aluminum-PPS interface and hence the strength of the joint.

A set of samples was also aged under natural outdoor weathering for six and 12 months. All of the joints retained more than 80% of their initial quasi-static strength. However, after one year of weathering, the ultimate lap shear strength of the joints pre-treated by SB and CC was reduced to 59% and 57% of their initial strength, respectively. The fracture surface of these joints showed that the humidity could penetrate inside the bonding area. In addition, a slight corrosion of the aluminum samples outside the bonding area may also contribute to the reduction in the strength of the joints. In the case of the SB and CC samples after 12 months of natural aging, it seems that the corrosion of the aluminum is the main reason for such a reduction in the joints’ strength. The joints pre-treated with PAA-P showed, however, a retained lap shear strength of more than 80% of their initial strength even after one year of weathering. Strong carbon-carbon chemical bonds and intimate contact between the joining parts are believed to significantly reduce the diffusion of moisture into the bonding area and increase the durability of the joints.

In summary, there are two mechanisms contributing to the reduction of the joints’ strength. In the case of accelerated aging for all the surface pre-treatments, penetration of humidity and hence the weakening of the interfacial bonds is the main weakening mechanism. Penetration of humidity inside the bonding area seems to remain the most important deterioration mechanism for all the joints after six months of natural aging. However, in the case of the SB and CC specimens after 12 months of natural aging, the predominant degradation mechanism changes to the weakening of the aluminum because of the occurrence of slight corrosion. For the PAA-P specimens, the main mechanism remains a slight penetration of humidity and the weakening of the bonding area.

It was also observed that natural aging had a more critical effect on the joints compared to accelerated aging. One reason was the fact that the precipitation contained chloride and other salty compounds, which are more detrimental to the aluminum and the interfacial bonds, rather than a pure humid environment. Furthermore, the free end of the joints in the natural aging experiments experienced (strong) wind that could influence the strength of the joints.

## Figures and Tables

**Table 1:** Average chemical composition (in wt %) of the SB and CC pre-treated AA2024-T3 surface after 28 days of accelerated aging by EDS analysis.

Surface Pre-Treatment	Al	O	C	N
SB	45.7	43.2	11.1	-
CC	63.3	32.4	0.8	3.5

**Table 2:** Average chemical composition (in wt %) of the SB and CC pre-treated AA2024-T3 surface before aging by EDS analysis.

Surface Pre-Treatment	Al	O	C	Cu	Mg
SB	87.3	6.8	1.2	3.1	1.6
CC	88.1	5.1	1.5	3.9	1.4

**Table 3:** Average chemical composition (in wt %) of the PAA and PAA-P pre-treated AA2024-T3 surface after 28 days of accelerated aging by EDS analysis.

Surface Pre-Treatment	Al	O	C	Cu	Mg	N	Cr	Sr
PAA	69.3	14.9	11.8	2.6	1.4	-	-	-
PAA-P	8.0	7.6	51.0	-	-	10.0	9.3	14.1

**Table 4:** Average chemical composition (in wt %) of the PAA and PAA-P pre-treated AA2024-T3 surface before aging by EDS analysis.

Surface Pre-Treatment	Al	O	C	Cu	Mg	P	S	Cr	Sr
PAA	76.9	14.9	2.0	3.0	1.5	1.7	-	-	-
PAA-P	0.2	17.8	76.0	-	-	-	-	2.5	3.5

**Table 5:** Average chemical composition (in wt %) of the flake-like features and agglomerates on the CF-PPS by EDS analysis.

Features	Al	O	C	Cu	Mg	S
Flake-like	42.4	32.1	-	0.5	0.5	24.5
Agglomerates	38.5	42.1	18.3	0.8	0.3	-
